# Occult Autoimmune Background for Epilepsy—The Preliminary Study on Antibodies Against Neuronal Surface Antigens

**DOI:** 10.3389/fneur.2021.660126

**Published:** 2021-10-21

**Authors:** Edyta Dziadkowiak, Helena Moreira, Katarzyna Buska-Mach, Magdalena Szmyrka, Sławomir Budrewicz, Ewa Barg, Marta Janik, Anna Pokryszko-Dragan

**Affiliations:** ^1^Department of Neurology, Wroclaw Medical University, Wroclaw, Poland; ^2^Department of Basic Medical Sciences, Wroclaw Medical University, Wroclaw, Poland; ^3^Euroimmun Poland, Wroclaw, Poland; ^4^Department of Rheumatology, Wroclaw Medical University, Wroclaw, Poland

**Keywords:** neuronal autoantibodies, epilepsy, connective tissue disorders, epileptogenesis, autoimmune reactivity

## Abstract

**Objective:** The objective of the study was to determine the incidence of antibodies against neuronal surface antigens (NSA-ab) in patients with different types of epilepsy, in comparison with the subjects diagnosed with immune-mediated disorders.

**Methods:** Forty patients with drug-resistant epilepsy (DRE) of unknown origin, 16 with post-stroke epilepsy, and 23 with systemic autoimmune disorders (SAD) with CNS involvement were included. NSA-ab were sought in serum using indirect immunofluorescence method. Relationships were analyzed between presence of NSA-ab and clinical presentation.

**Results:** NSA-ab was detected in the sera from five patients: anti-DPPX in one patient, anti-AMPAR1/R2 in two, anti-LGI1 in one and, in one case, both anti-CASPR2 and DPPX IgG. Out of these five patients, three represented the SAD subgroup and two the DRE subgroup. None of the patients with post-stroke epilepsy was positive for NSA-ab.

**Significance:** Autoimmune etiology is worth considering in patients with drug-resistant epilepsy of unknown origin. The presence of NSA-ab in patients with systemic autoimmune disorders may be caused by unspecifically enhanced autoimmune reactivity. NSA-ab seem not to be related to epilepsy resulting from ischemic brain injury.

## Introduction

Epilepsy affects up to 1.0% of the world population, and its effective management still constitutes a challenge. In recent years, there has been a significant expansion in our understanding of heterogeneous etiology of the epilepsy, underpinned by advances in modern neuroimaging and genetic testing. In 2017, the International League Against Epilepsy (ILAE) Classification of the Epilepsies recognized a range of etiologic groups, with autoimmune background regarded for the first time as an independent cause for epilepsy (1).

Systemic autoimmune diseases with involvement of the central nervous system (CNS) and epilepsy co-occur frequently. Some isolated autoimmune CNS disorders have been also identified with seizures as one of their core symptoms (2, 3). IgG autoantibodies against proteins on the neuronal surface (NSA-ab) are considered as main pathogens and markers in these disorders. NSA-ab include antibodies against voltage-gated potassium channel (VGKC) complex (leucine-rich glioma inactivated (1LGI1), contactin-associated protein-like 2 (CASPR2), N-methyl-D-aspartate receptor (NMDAR), gamma-amino-butyric B receptor (GABA_B_R), alpha-amino-3-hydroxy-5-methyl-4-isoxazolepropionic acid receptor (AMPAR), a subtype of glutamate receptor, dipeptidyl-peptidase-like protein 6 (DPPX), a regulatory protein of the Kv4.2 potassium channels (3, 4). Apart from NSA-ab, antibodies targeted against intracellular antigens (Hu, Ma, CV2, amphiphysin, Delta/Notch-like EGF-related receptor, Sox1, and glutamic acid decarboxylase) may also account for a specific group of immune-mediated CNS orders, usually of paraneoplastic origin and infrequently associated with epileptic seizures (5). Currently, the antibodies that target molecules on the surface of neurons seem most relevant in epileptology, although the full spectrum of autoantibody-related epilepsies is yet to be understood (6). Other autoantibodies target intracellular antigens, such as those directed against the enzyme glutamic acid decarboxylase (GAD) and onconeuronal antibodies.

The process of epileptogenesis may be mediated by NSA-ab through different pathways. They are hypothesized to mediate neuronal dysfunction (via ion channels and/or membrane receptors activity) or to be directly involved in inflammatory or ischemic damage to the cerebral structures (4, 6, 7). It is also presumed that these antibodies rather act as markers of the pathological process than actively contribute to CNS injury. Better recognition of putative autoimmune background for seizures and identification of the patients probable to have such an etiology of epilepsy seems substantial to optimize the diagnostic and treatment strategies.

In view of that, we aimed at investigating incidence of NSA-ab comparatively in the groups of patients with epilepsy of unknown origin, with symptomatic post-stroke epilepsy, and with systemic autoimmune disorders affecting CNS. Our second goal was to analyze relationships between the presence of NSA-ab and clinical presentation.

## Materials

The study comprised 79 patients (35 men, 44 women, average aged 45, 6 years), hospitalized or consulted at the Department of Neurology, Wroclaw Medical University, in the years 2018–2019, who were selected on the basis of the documented diagnosis and course of the disease.

Three specific subgroups of patients were determined as follows:

1. The patients diagnosed with drug-resistant epilepsy (DRE) of unknown origin. DRE was recognized, according to the ILAE definition, based on failure to achieve sustained seizure freedom with adequate trials of two well-tolerated and appropriately chosen and used AED schedules (whether as monotherapies or in combination) (8). Exclusion criteria in this subgroup included structural changes in the brain in magnetic resonance (MRI) and a history and evidence of infectious, immune-mediated, metabolic disorders or substance abuse as potential background for epilepsy.
2. The patients with post-stroke epilepsy (PSE), defined as two or more unprovoked epileptic seizures following stroke, which occurred more than 2 weeks after its acute phase, and in the absence of other obvious causes or a history of prestroke epilepsy (9–11). Patients with PSE and stroke in the course of systemic autoimmune disease were assigned to subgroup 3.
3. The patients diagnosed with immune-mediated systemic autoimmune disorders (SAD) with symptoms and signs of the nervous system involvement. SAD comprised systemic lupus erythematosus (diagnosed according to SLICC 2012 classification criteria) (12), antiphospholipid syndrome (Sydney classification criteria) (13), primary Sjogren syndrome (14), and undifferentiated connective tissue disease (UCTD)—disorders that share clinical and serological manifestations with definite SAD but do not fulfill any of the specific classification criteria (15).

## Characteristics of the Studied Subgroups

The age and gender structure of numbers of patients assigned to subgroups (1)–(3), are presented in the Table 1.

**Table 1:** Age and gender structure of the studied subgroups.

**Diagnosis**	**Patients**			
	**Female**	**Male**	**Age range (years)**	**Median**
(1) Drug-resistant epilepsy (*n* = 40)	17	23	18–79	38.5
(2) Post-stroke epilepsy (*n* = 16)	8	8	50–85	66
(3) Systemic autoimmune disorders (*n* = 23)	19	4	22–60	39

### Drug-Resistant Epilepsy

The classification of the seizures according to standardized diagnostic (revised) guidelines by the International League Against Epilepsy. Five patients had more than one type of seizure. Table 2 shows the distribution of patients by epilepsy type.

**Table 2:** Types of epilepsy in subjects with drug-resistant epilepsy.

Focal	20
Generalized	3
Combined focal and generalized	17

Duration of DRE ranged from 6 to 16 years (median of 7.4 years). Having experienced three to five failed attempts of anti-epileptic treatment regimen, all the patients with DRE were currently undergoing polytherapy with two or three AEDs (Table 3).

**Table 3:** AED combinations in patients with DRE.

**Combination of polytherapy**	**Patients**
OXC + LCM	10
LEV + LTG	12
BRV + LTG	5
VPA + LTG	3
TGB + ESX + AZZ	4
LCM + LEV + ESX	3
CBZ + TPM	3

*AAZ, acetazolamide; BRV, brivaracetam; CBZ, carbamazepine; ESX, ethosuximide; LCM, lacosamide; LTG, lamotrigine; LEV, levetiracetam; OXC, oxcarbazepine; TGB, tiagabine; TPM, topiramate; VPA, valproic acid; DRE, drug-resistant epilepsy*.

### Post-stroke Epilepsy

This subgroup involved 16 patients: 13 with previous ischemic stroke and 3 with previous hemorrhagic one, confirmed with CT scan. All the strokes were located in the cerebral cortex: hemorrhagic ones—in the right hemisphere, ischemic strokes—in the left (11 cases) or right (5 cases) hemisphere.

In 56% (9/16 patients), seizures occurred within 12 months after the stroke, and in the remaining 7/16 patients, they occurred within a longer period. Duration of PSE ranged from 2 to 38 years (mean: 6.0 years). Patients, 7/16, had focal seizures (Table 4).

**Table 4:** Types of epilepsy in subjects with post-stroke epilepsy.

Focal	15
Generalized	0
Combined focal and generalized	1

All the patients with PSE were undergoing monotherapy: in 12 cases with levetiracetam, in 3 with carbamazepine, and in 1 with sodium valproate.

### Immune-Mediated Disorders

Twenty-three patients were diagnosed with SAD-−12 with systemic lupus erythematosus, 8 with systemic lupus erythematosus and antiphospholipid syndrome (APS), 1 with systemic lupus erythematosus and secondary Sjogren syndrome, and 2 patients with undifferentiated connective tissue disease. Neuropsychiatric manifestations included cognitive impairment, TIA/strokes, transverse myelitis, polyneuropathy, tension headache, depression, and psychosis. EEG was abnormal in 12 patients. All the patients were undergoing immunosuppressive treatment.

Written informed consent was obtained from all patients prior to their participation in the study. The study protocol was approved by the Bioethics Committee at Wroclaw Medical University.

## Methods

In all the patients, presence of NAbs was investigated using the IIFT Autoimmune Encephalitis Mosaic 6 test (EUROIMMUN), BD Vacutainer® Plus Plastic Serum Tubes (BD, Poland). In the patients from subgroups 1 and 2, blood sample was collected at least 24 h after a recent epileptic seizure, and in the subjects with IME, it was collected prior to the first infusion of intravenous immunoglobulins.

### Blood Sample

Blood was collected into BD Vacutainer® Plus Plastic Serum Tubes (BD, Poland). The blood samples were centrifuged at 500 × *g* for 15 min. The serum was transferred to a polypropylene tube and stored at 4°C until the time of analysis. For longer storage, the serum samples were frozen at −20°C. The analysis was routinely carried out within 2 weeks of blood collection.

### Evaluation of Antineuronal Antibodies in Serum

The presence of human IgG immunoglobulins against neuronal surface antigens in the serum of patients was assessed by indirect immunofluorescence methods using the IIFT Autoimmune Encephalitis Mosaic 6 test kit (EUROIMMUN, Poland). The test enables qualitative or semiquantitative determination of six antineuronal antibodies against glutamate receptor—type NMDA and type AMPA1/2, CASPR2, LGI1, DPPX, and GABAB receptors (GABARB1/B2).

Positive samples were re-analyzed at a 1:100 dilution. The results were evaluated as positive or negative.

In the patients seropositive for NSA-ab, clinical presentation and other diagnostic findings were thoroughly analyzed.

## Results

**Figure 1:**
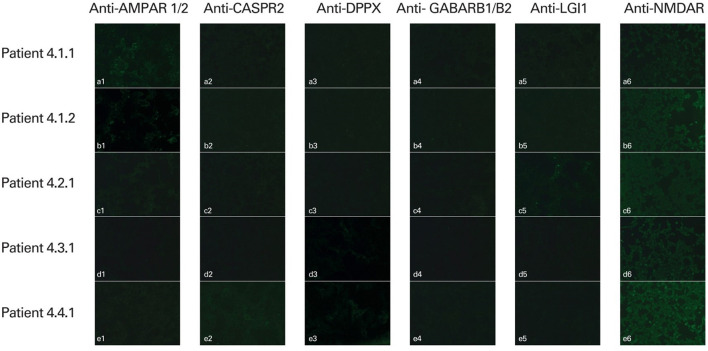
Detection of human antineuronal antibodies in the serum of patients using indirect immunofluorescence methods (IIFT Autoimmune Encephalitis Mosaic 6 test, EUROIMMUN, Poland). Slides were evaluated using fluorescence microscopy with excitation filter 450–490 nm, color separator 510 nm, blocking filter 515 nm, and optical magnification of × 400 (AMPAR 1/2, CASPR2, DPPX, GABARB1/B2, LGI1; ×200 (NMDAR). Positive staining: **(a1, b1)** anti-AMPAR1/R2 positive; **(c5)** anti-LGI1 positive; **(d3)**, **(e3)** anti-DPPX positive; **(e2)** anti-CASPR2 positive. Negative reaction: **(a2–a6**, **b2–b6**, **c1–c4**, **c6**, **d1–d2**, **d4–d6**, **e1**, **e4–e6)**.

NSA-ab was detected in the sera from five (6, 3%) patients: in two patients anti-AMPAR1/R2, in one patient anti-CASPR2, in two patients anti-DPPX, in one patient anti-LGI1. In one case, both anti-CASPR2 and DPPX IgG were found. Out of these five patients, three represented subgroup 3 (diagnosed with SLE) and two represented subgroup 1 (DRE). None of the patients with PSE (subgroup 2) was positive for the studied Ig (Figure 1).

The clinical characteristics of the patients positive for NSA-ab were as follows.

### Anti-AMPAR1/R2 Positive

This is a case of a 62-year-old man with SLE (predominant kidney involvement) and secondary antiphospholipid syndrome. At 48 years of age, he suffered from ischemic stroke and toe ischemia, and his immunological markers included ANA at a titer of 1:3,200 specified as anti-dsDNA, anti-Ro52, anti-RNP, and anti-CENP. He was positive for lupus anticoagulant; other antiphospholipid antibodies were not detected. Eight years later, he was diagnosed with epilepsy (focal seizures with motor onset and preserved awareness) and effectively treated with VPA (the last epileptic seizure occurred at 57 years of age). Brain MRI showed multiple vascular lesions within the subcortical white matter and cerebral cortex of both hemispheres, with segmental cortical atrophy. The EEG recording was normal. He was currently being treated with prednisone, azathioprine, chloroquine, and acenocoumarol.

This is a case of a 69-year-old woman with SLE and secondary antiphospholipid syndrome. She was diagnosed with lupus at the age of 50. Her SLE manifestations included arthralgia, myalgia, pleuritis, anemia, leukopenia, and kidney involvement. She had a high titer of ANA (anti-dsDNA and anti RNP)-−1:10,000, low levels of complement components C3 and C4. She was positive for lupus anticoagulant; other antiphospholipid antibodies were not detected. At the age of 54, she suffered from ischemic stroke in the right cerebellar hemisphere, without permanent neurological deficit. No epileptic seizures had been observed. The EEG recordings were normal. Clinical and electrophysiological features of polyneuropathy were found.

The patient was being treated with prednisone, chloroquine, and acenocoumarol.

### Anti-LGI1 Positive

This is a case of a 57-year-old woman with SLE and secondary Sjogren syndrome. She was diagnosed at the age of 41 and presented with arthralgia, rash, photosensitivity, pleuritis, and sicca syndrome. She was positive for ANA at a titer of 1:1,000, characterized as anti-dsDNA, anti-Ro 52, anti-Ro/SSA, and anti-La/SSB. Complement levels were low, and antiphospholipid antibodies were not detected. From the age of 43, she also developed fibromyalgia. Her neuropsychiatric symptoms included cognitive impairment and depression, without history of seizures. The EEG recordings were normal. The MRI showed multifocal cortical infarcts in the both frontal and parietal lobes. She had paresthesia of the limbs, but the nerve conduction studies and electromyography showed no abnormalities. The patient was being treated with methotrexate, hydroxychloroquine, and methylprednisolone.

### Anti-DPPX Positive

This is a case of an 18-year-old man suffering from DRE of unknown origin. Epilepsy was diagnosed at the age of 13 and defined as drug-resistant at the age of 16. Seizures (generalized onset tonic–clonic) occurred twice a month. The MRI of the brain was normal. The normal CSF result was obtained. In an EEG recording, interictal epileptiform discharges (IEDs) as bilateral spikes and slow waves were observed, with normal background activity.

### Anti-CASPR2 and Anti-DPPX Positive

This is a case of a 60-year-old man suffering from DRE of unknown origin. Epilepsy was diagnosed at the age of 45 and defined as drug-resistant at the age of 58. Two types of seizures occurred: tonic–clonic ones with generalized onset and focal ones with non-motor (emotional) onset and preserved awareness. In addition, the patient was diagnosed with mixed personality disorder. The MR of the brain was normal. The normal CSF result was obtained. In EEG recordings, generalized (predominantly left) slowing (delta waves) of background activity was observed, without paroxysmal discharges.

## Discussion

The relationships between autoimmune mechanisms and epilepsy remain unclear. The seizures are likely to be caused by a variety of mechanisms, including specific and non-specific immunity, neuronal damage, and ischemia (4, 7, 8). It is still a matter of debate, whether epilepsy may be the only clinical manifestation of autoimmune encephalitis or whether immune-mediated mechanisms may contribute to development of seizures following previous structural damage to the brain (1, 2, 16, 17). The design of our preliminary study (especially the structure of the studied subgroups) was meant to address the above theories.

In our study, NSA-ab was not found in any patient with post-stroke epilepsy. It can be therefore supposed that these antibodies do not mediate epileptogenesis in the case of structural changes in the brain (at least specifically in cerebrovascular disease). Elisak et al. (18) explored the prevalence of neural-surface antibodies and antibodies against glutamic acid decarboxylase (GAD) in patients with chronic temporal lobe epilepsy (TLE). Significant levels of antibodies were detected in 8 out of 163 (5%) TLE patients, while five patients had uni- or bilateral temporal lobe lesions in MRI, and three patients had not. However, seropositive subjects had some immune-mediated comorbidities (autoimmune thyroiditis, type 1 diabetes). In six of these patients, immunotherapy was applied, and in three, it resulted in reduction of seizures. The study from Vanli-Yavuz et al. (19) showed the presence of neuronal autoantibodies in 22.5% of the patients with mesial temporal lobe epilepsy with hippocampal sclerosis (MTLE-HS) and found that advanced neuroimaging findings in the extra/temporal region were significantly more frequent in the seropositive group. Ekizoglu et al. (20) reported a similar prevalence of NSA-ab (23%) in the patients with MTLE-HS, while Nóbrega-Jr et al. (21) found NSA-ab in none of 100 patients with this diagnosis. It should be highlighted that the causative relationships between mesial temporal sclerosis and epilepsy still remain unclear), including the hypothesis of hippocampal sclerosis resulting from the evolution of limbic emcephalitis (20–22). According to the definition of post-stroke epilepsy, in our PSE subgroup, possible contributing comorbidities or prestroke epilepsy had been excluded, so seizures were definitely following vascular damage to the brain.

Subjects, 2 out of 40, with drug-resistant epilepsy of unknown origin were positive for NSA-ab. Gozubatik-Celik et al. (23) demonstrated the presence of neuronal autoantibodies in ca. 14% patients with focal epilepsy of unknown cause (Abs against VGKC-complex, VGCC, GAD, LGI1, CASPR2, NMDA, AMPA, and GABAB receptors), and a similar frequency (up to 20%) for epilepsy with unknown origin was reported by other authors (18, 20, 24). Recognition of immune-mediated background in cases of unknown origin of epilepsy, especially a drug-resistant one, is crucial because it allows application of targeted and possibly efficacious immunotherapy. Treatment with intravenous immunoglobulins was shown to reduce frequency of seizures in drug-resistant cases of epilepsy of various etiologies (25, 26). Especially, the subjects with Abs against neuronal cell-surface proteins are expected to respond well to such treatment (24, 27).

Seizures may occur as a clinical manifestation of autoimmune encephalitis, but are usually accompanied by other typical symptoms: cognitive decline, psychiatric disorders, and disturbed consciousness, and often resolve after effective treatment of encephalitis (7, 28–37).

Except for seizures, both our patients with anti- DPPX and anti-CASPR2 Abs did not develop other clinical presentation typical for encephalitis. It is suggested that in a small percentage of patients with drug-resistant epilepsy, seropositive for NSA-ab, other subtle signs of CNS involvement can be revealed after a thorough search (3). Furthermore, to verify the diagnosis of encephalitis, the presence of NSA-ab should be confirmed in CSF. In our patients, general CSF evaluation conducted at the onset of epileptic seizures (from 6 to 16 years before entering this study) revealed correct general examination of the cerebrospinal fluid; virological tests were negative. After obtaining the current results of NSA-ab in the serum, we offered these two patients specific CSF testing, but they did not give their consent to lumbar puncture.

It is worth highlighting, that three out of five patients with confirmed presence of NSA-ab were diagnosed with SLE, although only two of them presented with clinical symptoms of CNS involvement. Possible background of epilepsy in systemic autoimmune disorders may be associated with autoimmune inflammation, which crosses the blood–brain barrier and directly affects cerebral neurons, or secondary damage to the brain due to ischemic injury or calcifications (celiac disease, SLE) (38, 39). SLE is the prototype of SAD that can involve the complete spectrum of psychiatric and neurological disorders. Epilepsy may occur in 2.1–11.6% of SLE patients (4, 37). It has been shown to be associated with stroke in the course of SLE and with the presence of antiphospholipid antibodies (APLA): anti-cardiolipin (aCL), lupus anticoagulants (LA), and anti-beta 2 glycoprotein 1 (35–38). APLA, apart from causing microvascular thrombosis and ischemic damage to the brain, is supposed also to react directly with neuronal cells and contribute to their dysfunction, including epileptogenesis (35). However, patients with neuropsychiatric manifestation of SLE (NPSLE) were demonstrated to have elevated levels not only of APLA but also anti-ribosomal P Abs and anti-neuronal Abs (36, 38). In addition, Hirohata et al. (40) found antibodies against the linear epitope in the NR2 subunit of the NMDA receptor in the sera of 61% of patients with NPSLE (35, 38, 41). Anty-NMDAR has been also shown to be part of the anti-dsDNA antibodies. Furthermore, there is some evidence in the literature for co-occurence of NSA-ab in other inflammatory CNS disorders, e.g., the presence of anti-NMDAR, anti-VGKC or anti-GlyR Abs in the patients with sporadic forms of Creutzfeld–Jacob disease (33, 42, 43), and anti-NMDAR antibodies in patients with herpes encephalitis, or in those with previous HSV-1 infection It has been hypothesized that inflammatory brain injury exposes NMDARs and triggers autoimmune response against them (40, 44–49).

There are a few hypotheses explaining the co-occurence of different types of autoAbs, irrespective of clinical presentation. First, cross-linking and internalization is a common mechanism for autoantibodies targeting ionotropic receptors (4, 5, 28). Second, due to cross-reactivity or molecular mimicry, autoimmune response may be triggered against neuronal antigens parallel to the other ones (45, 46). Finally, increase in humoral response not specific for a particular systemic autoimmune disease may result from general upregulation of the immune system (8, 17).

Our study has some limitations, including relatively small sample size and heterogeneity of the group with immune-mediated disorders, which might have affected the results. Another limitation was the determination of autoAbs only in the serum, without CSF testing. Therefore, the findings have to be interpreted very cautiously. However, the study was meant to provide some preliminary results, supporting and giving direction to further investigations. To allow a better insight in a concept of immune-mediated background for epilepsy, these should include a sensitive and specific method of testing a wide panel of NSA-ab in the serum and CSF conducted in carefully selected groups of epileptic patients.

## Conclusions

Despite a small percentage of NSA-ab-positive patients in the study group, autoimmune etiology is worth considering in patients with drug-resistant epilepsy of unknown origin. The presence of NSA-ab in patients with systemic autoimmune disorders, especially without typical clinical manifestation, may be caused by unspecifically enhanced autoimmune reactivity. NSA-ab seem not to be related to epilepsy resulting from ischemic brain injury.

## Data Availability Statement

The original contributions presented in the study are included in the article/supplementary material, further inquiries can be directed to the corresponding author/s.

## Ethics Statement

The studies involving human participants were reviewed and approved by Bioethics Committee of Wroclaw Medical University. The patients/participants provided their written informed consent to participate in this study.

## Author Contributions

ED conceptualized and designed the study. ED and HM drafted the manuscript. ED, KB-M, MS, SB, EB, MJ, and AP-D critically revised the manuscript for important intellectual content. ED, HM, KB-M, and MS collected the data. All authors contributed to the article and approved the submitted version.

## Funding

This study was supported by Wroclaw Medical University SUB.C.220.21.028.

## Conflict of Interest

KB-M and MJ was employed by company Euroimmun Poland. The remaining authors declare that the research was conducted in the absence of any commercial or financial relationships that could be construed as a potential conflict of interest.

## Publisher's Note

All claims expressed in this article are solely those of the authors and do not necessarily represent those of their affiliated organizations, or those of the publisher, the editors and the reviewers. Any product that may be evaluated in this article, or claim that may be made by its manufacturer, is not guaranteed or endorsed by the publisher.

## Abbreviations

NSA-ab: antibodies against neuronal surface antigens; mesial temporal lobe epilepsy with hippocampal sclerosis
SAD: systemic autoimmune disorders
SLE: systemic lupus erythematosus
NPSLE: neuropsychiatric systemic lupus erythematosus.
